# Hypodermic Injections of Ether in the Treatment of Neuralgia

**Published:** 1890-09

**Authors:** 


### HYPODERMIC INJECTIONS OF ETHER IN THE TREATMENT OF NEURALGIA.

The parenchymatous injection of ether for the relief of neuralgia is by no means a new method of treatment, though it has had but few advocates, and has been practiced to only a limited extent. Kumps (*Revue de Therapeutique*, June 15, 1890) again brings the method forward as a valuable addition to the therapeutics of neuralgia, and cites a number of cases in which relief followed the injection of equal parts of alcohol and ether into the neighborhood of the affected nerve. Among other cases reported by him are the following:

A neuralgia of the shoulder which for two weeks had prevented sleep and had resisted a variety of other methods of treatment, was cured by a single injection. Two cases of sciatica, both of which were much relieved after the first injection. Two cases of facial neuralgia, and three of rheumatic neuralgia of the head were also relieved.

Kumps uses the injections not only in cases of true neuralgia, but in rheumatic pains and in torticollis. Two cases of the latter disease were cured by the method, only one injection being required in each case. The amount of the mixture used by the author is 30 minims, and he states that though the pain of the injection is considerable it soon passes away. There is also often some local swelling which can be dissipated by gentle manipulation.—*London Medical Recorder*, July, 1890.

